# Multiple indicator cluster survey (MICS) related to early childhood health: an ecological intercontinental analysis

**DOI:** 10.1515/med-2026-1495

**Published:** 2026-10-02

**Authors:** Evilly Santos Andrade, Nuno Damácio de Carvalho Félix, Martha Peixoto da Silva, Felipe de Alcântara Moreira, Maria Carolina Ortiz Whitaker, Simão Pedro Pereira Vilaça, Alan Lins Fernandes, Amâncio António de Sousa Carvalho, Climene Laura de Camargo, Josielson Costa da Silva

**Affiliations:** Postgraduate Program in School of Nursing and Health (PPGENF), Federal University of Bahia (UFBA), Salvador, Brazil; School of Nursing, University of Minho (U.minho), Braga, Portugal; Postgraduate Program in Medicine and Health (PPgMS), Federal University of Bahia (UFBA), Salvador, Brazil; Department of the School of Health, University of Trás-os-Montes e Alto Douro (UTAD), Vila-Real, Portugal; Research Centre on Child Studies, University of Minho (U.Minho), Braga, Portugal

**Keywords:** child health, developing countries, health surveys, United Nations, child development, Human Development Index-HDI

## Abstract

**Objectives:**

Child health is a fundamental measure of a population’s social and sanitary conditions and enables the identification of priority challenges to promote well-being in early childhood. This study aimed to conduct a joint and comparative assessment of reported child health indicators in developing countries participating in the sixth round of the Multiple Indicator Cluster Surveys (MICS6).

**Methods:**

This ecological cross-sectional study was conducted from April to July 2024, using UNICEF data from developing countries that implemented MICS6 and reported country-level continuous quantitative indicators, such as under-five mortality, complete vaccination coverage, adequate skilled childbirth care, prevalence of low birth weight, and access to safe drinking water services. The Human Development Index (HDI) was included as an additional indicator. Statistical analyses comprised multiple linear regression and Pearson’s correlation.

**Results:**

Of the 72 countries and territories initially considered, 58 were included in the inferential and descriptive analyses, and 39 had complete data and were included in the visual mapping, representing the following regions: Africa (n=17), Asia (n=13), Oceania (n=5), Central America (n=2), South America (n=1) and Europe (n=1). Access to safe drinking water (*β* = −0.63; 95 % CI: −1.11 to −0.14; p=0.013) and HDI (*β* = −101.18; 95 % CI: −175.03 to −27.32; p=0.008) were significantly and inversely associated with under-five mortality risk, as were the strong inverse correlations between mortality and both HDI (r = −0.793) and access to safe drinking water (r = −0.782). All correlations among variables were statistically significant (p<0.001). Africa exhibited the poorest performance across multiple child health indicators, while Asia reported the highest prevalence of low birth weight. Serbia, the sole European country analyzed, demonstrated the most favorable outcomes overall.

**Conclusions:**

These findings underscore substantial inequalities in child health, particularly in Africa, and highlight the imperative for targeted global health policies to address these disparities.

## Introduction

The Multiple Indicator Cluster Surveys (MICS), initiated by the United Nations International Children’s Emergency Fund (UNICEF) in the 1990s, are globally standardized household surveys conducted in partnership with national governments and international organizations [1]. These surveys provide valid, comparable data that are instrumental in shaping evidence-based policies across health, education, and social protection, ultimately advancing the well-being of women and children worldwide [2].

Health and social interventions informed by MICS data have been pivotal in addressing persistent childhood health challenges such as low birth weight [3], under-five mortality [4], incomplete vaccination, inadequate skilled childbirth care, and limited access to safe drinking water [5]. Complementarily, the Human Development Index (HDI), which measures life expectancy, education, and income per capita, serves as a key composite indicator encompassing these social determinants and health outcomes [4], 6].

The systemic use of these measures facilitates identification of vulnerable populations and underpins targeted public policies. Rebouças et al. [7] emphasize the deep historical roots of social inequalities affecting child health, highlighting commonly used indicators including infant and under-five mortality, low birth weight, prematurity, stunting, and overweight. Under-five mortality is a key public health indicator, highly responsive to preventive interventions, and globally recognized as the most definitive and comprehensive proxy for overall child health and survival [8], 9]. While global efforts have made strides in reducing mortality rates in this age group, significant disparities persist, particularly in Sub-Saharan Africa [8]. Inequalities linked to socioeconomic factors such as low parental education and household income continue to negatively affect child development, although protective factors like breastfeeding can mitigate these effects [10].

Prior studies have examined individual health indicators – such as maternal and child nutrition [11], 12], infant/child mortality [13], [14], [15], vaccination coverage [16], 17], skilled birth attendance [18], 19], low birth weight [20], 21], and inadequate access to safe drinking water [22], 23] – , althoughintegrated analyses combining data from the sixth round of the MICS with the HDI remain limited. Therefore, this study was designed based on the rationale that child health outcomes reflect not only specific health interventions, but also broader socioeconomic and development contexts captured by the HDI. Furthermore, the present study seeks to understand how human development levels, vaccination coverage, skilled childbirth care, low birth weight, and access to safe drinking water predict the under-five mortality risk among developing countries children under five years of age in developing countries participating in MICS6.

We hypothesized that countries with higher levels of human development would present more favorable profiles of under-five mortality, vaccination coverage, skilled childbirth care, low birth weight, and access to safe drinking water compared with countries with lower HDI. Thus, the research question guiding this study was: “How do human development levels, vaccination coverage, skilled childbirth care, low birth weight, and access to safe drinking water predict the under-five mortality risk, and how are all these indicators distributed among developing countries participating in MICS6?”

## Methods

### Study design

This ecological cross-sectional study was conducted from April to July 2024, using secondary data from the MICS6 (UNICEF). The MICS6 surveys included developing countries from five continents, offering standardized, valid, and comparable data on child health indicators [1].

### Population and sample

Data collection was conducted directly via the UNICEF, utilizing public and non-overlapping data previously published from the most recent MICS round (MICS6), focused on children under five years of age (Table 1 and Supplementary Figure 1). The inclusion criteria required countries and territories to have a research status marked as “completed” and to have data collected on the following country-level continuous quantitative indicators relevant to this study: under-five mortality rate, complete vaccination coverage, prevalence of severe low birth weight, adequate skilled childbirth care, and access to safe drinking water services. Additionally, the HDI for MICS6 countries was retrieved to complement the analysis. Countries and territories with at least 50 % of the required indicators or HDI data were included in the inferential and descriptive analyses; however, those lacking complete data were excluded from the visual mapping to ensure comparability.

**Table 1:** Reported indicators of under-five mortality, complete vaccination coverage, adequate skilled childbirth care, prevalence of low birth weight, and basic access to safe drinking water services in developing countries participating in MICS6 included in regression (n=58). Descriptive data expressed as mean (SD), No. (%), or absolute values.

	Human development index	Under-five mortality	Adequate skilled childbirth care	Complete vaccination coverage	Prevalence of low birth weight	Access to safe drinking water services
**Africa (n=19), mean** ± **SD**	0.536 ± 0.081	62.4 ± 29.6	77.6 ± 19.8	55.2 ± 23.0	3.6 ± 2.8	67.0 ± 18.8
Malawi	0.512	56.0	96.4 %	72.6 %	2.6 %	74.2 %
Nigeria	0.535	102.0	50.7 %	44.0 %	–	41.5 %
São Tomé and Príncipe	0.618	14.0	96.8 %	41.5 %	1.4 %	77.2 %
Chad	0.394	122.0	47.2 %	–	11.1 %	50.9 %
Comoros	0.558	36.0	97.2 %	57.0 %	1.9 %	91.9 %
Eswatini	0.597	41.0	93.4 %	77.0 %	1.3 %	78.2 %
Benin	0.525	80.0	80.8 %	27.6 %	5.9 %	72.2 %
Tunisia	0.576	17.0	99.5 %	79.1 %	0.6 %	85.9 %
Central African Republic	0.404	99.0	42.9 %	13.2 %	7.0 %	37.5 %
Guinea-Bissau	0.483	51.0	53.8 %	19.3 %	3.8 %	59.8 %
Madagascar	0.501	59.0	46.0 %	33.0 %	6.0 %	41.0 %
Lesotho	0.514	76.2	86.6 %	68.8 %	2.5 %	79.4 %
Gambia	0.500	57.0	82.7 %	83.2 %	2.7 %	84.8 %
Zimbabwe	0.593	65.0	86.0 %	84.5 %	1.6 %	63.6 %
Algeria	0.745	19.0	98.8 %	61.0 %	0.8 %	94.1 %
Togo	0.539	71.0	69.4 %	48.5 %	2.8 %	68.1 %
Ghana	0.632	56.0	78.9 %	78.1 %	2.4 %	79.4 %
Sierra Leone	0.477	94.0	81.6 %	69.4 %	3.7 %	59.5 %
Democratic Republic of the Congo	0.479	70.0	85.2 %	35.0 %	7.1 %	33.2 %
**Asia (n=18), mean** ± **SD**	0.634 ± 0.103	34.6 ± 16.2	83.5 ± 17.3	65.4 ± 22.7	5.1 ± 5.3	90.2 ± 9.5
Pakistan (Sindh)	0.544	46.0	82.7 %	43.8 %	18.1 %	90.4 %
Pakistan (Punjab)	0.544	69.0	76.4 %	76.5 %	6.4 %	95.9 %
Pakistan (Balochistan)	0.544	53.0	49.9 %	26.9 %	11.6 %	79.6 %
Pakistan (Khyber Pakhtunkhwa)	0.544	39.0	76.5 %	45.8 %	7.1 %	86.8 %
Vietnam	0.703	14.0	96.1 %	78.6 %	–	97.8 %
State of Palestine	0.715	14.2	99.7 %	86.0 %	0.6 %	99.2 %
Bangladesh	0.661	40.0	59.0 %	–	5.2 %	98.0 %
Nepal	0.602	28.0	77.2 %	70.2 %	6.5 %	95.4 %
Thailand	0.800	–	99.6 %	82.6 %	1.8 %	99.6 %
Turkmenistan	0.745	38.0	100.0 %	–	0.5 %	99.9 %
North Korea	–	15.0	99.5 %	97.3 %	1.8 %	93.2 %
Lao People’s Democratic Republic	0.607	46.0	64.4 %	48.1 %	4.9 %	–
Iraq	0.686	26.0	95.6 %	58.1 %	1.0 %	85.7 %
Mongolia	0.739	19.0	99.3 %	86.9 %	0.5 %	82.5 %
Kyrgyzstan	0.692	20.0	99.8 %	76.7 %	0.4 %	92.6 %
Uzbekistan	0.727	25.0	99.2 %	94.0 %	0.4 %	95.0 %
Yemen	0.455	41.0	60.9 %	38.3 %	14.7 %	72.8 %
Afghanistan	0.478	55.0	67.5 %	36.6 %	5.6 %	68.8 %
**Europe (n=6), mean** ± **SD**	0.803 ± 0.022	12.5 ± 4.9	99.6 ± 0.5	83.9 ± 3.7	0.6 ± 0.7	97.5 ± 2.8
Kosovo	–	16.0	99.5 %	88.1 %	0.2 %	92.1 %
Montenegro	0.832	–	98.8 %	–	1.9 %	99.1 %
North Macedonia	0.770	–	100.0 %	82.5 %	0.2 %	98.8 %
Georgia	0.802	–	–	–	0.3 %	96.5 %
Belarus	0.808	–	99.9 %	–	–	99.4 %
Serbia	0.802	9.0	99.9 %	81.0 %	0.2 %	99.1 %
**Central America (n=7), mean** ± **SD**	0.747 ± 0.072	22.2 ± 7.1	98.4 ± 2.2	69.9 ± 14.9	0.5 ± 0.4	97.3 ± 2.3
Jamaica	0.709	–	–	88.6 %	–	93.1 %
Trinidad and Tobago	0.810	15.0	98.8 %	55.1 %	–	99.4 %
Honduras	0.621	21.0	94.1 %	77.6 %	1.2 %	95.7 %
Costa Rica	0.809	–	98.5 %	73.1 %	0.3 %	99.6 %
Cuba	0.764		100.0 %	48.9 %	0.2 %	97.1 %
Dominican Republic	0.767	32.0	99.2 %	62.1 %	0.6 %	98.4 %
Turks and Caicos Islands	–	21.0	100.0 %	84.0 %	0.3 %	98.0 %
**Oceania (n=5), mean** ± **SD**	0.689 ± 0.054	28.0 ± 19.7	95.7 ± 5.0	65.2 ± 30.5	1.1 ± 0.6	95.1 ± 7.9
Fiji	0.730	18.0	99.8 %	95.4 %	1.2 %	97.6 %
Tonga	0.745	11.0	98.3 %	93.5 %	0.3 %	99.0 %
Samoa	0.707	20.0	88.9 %	26.8 %	1.4 %	98.6 %
Tuvalu	0.641	30.0	99.5 %	68.3 %	0.7 %	99.4 %
Kiribati	0.624	61.0	91.9 %	42.1 %	2.0 %	81.0 %
**South America (n=3), mean** ± **SD**	0.762 ± 0.070	22.0 ± 4.2	98.3 ± 0.6	49.2 ± 30.2	1.5 ± 1.0	97.2 ± 2.4
Argentina	0.842	–	98.8 %	–	1.1 %	99.4 %
Guyana	0.713	25.0	97.6 %	70.6 %	2.7 %	94.6 %
Suriname	0.730	19.0	98.4 %	27.9 %	0.8 %	97.5 %

### Data sources and procedures

The MICS6 consists of nationally representative household surveys conducted by UNICEF’s Global MICS Team between 2017 and 2023. Designed by a specialized technical group, the surveys employ rigorously developed, modular questionnaires to collect reliable and comparable data on child and maternal health worldwide [2].

The MICS sampling methodology starts with countries selecting key indicators, defining reporting domains, and setting precision targets to estimate sample size. These initial estimates are balanced against implementation factors such as costs, fieldwork duration, and time constraints to determine final sample sizes. The protocol prohibits replacing sampled clusters or households and limits household numbers per cluster to optimize survey efficiency, ensuring both statistical rigor and practical feasibility, as detailed in previous publications [24].

Survey implementation follows a multi-stage sampling design beginning with the selection of Enumeration Areas (EAs) from an up-to-date sampling frame – usually the national census – using systematic probability proportional to size (PPS) based on household counts. Households within selected EAs are then listed and chosen through random systematic sampling. Finally, specific individuals, such as a randomly selected child under five, are chosen within households. Selection probabilities are recorded at each stage to calculate sampling weights adjusted for non-response, ensuring representative data [24], 25].

Herein, the secondary data were compiled into an electronic spreadsheet, with each selected indicator systematically separated. Median values and extremes (minimum and maximum) were identified and arranged in ascending order, facilitating data standardization and preparation for subsequent comparative analyses.

### Health indicators and social determinants

The health indicators and social determinants in MICS6 [2], 26], along with the HDI [27], included:

1. Under-five mortality: defined as the number of deaths of children under five per 1,000 live births, reported by mothers;
2. Complete vaccination coverage: determined by whether children under five have received all recommended vaccine doses, verified by vaccination cards or caregiver report;
3. Adequate skilled childbirth care: the proportion of births attended by skilled health personnel, based on maternal recall;
4. Prevalence of low birth weight: calculated from birth weight data with a cutoff of less than 2,500 g;
5. Access to safe drinking water services: assessed by WHO/UNICEF criteria based on water source and treatment practices;
6. The Human Development Index (HDI): a composite index measuring health, education, and living standards, sourced from the United Nations Development Programme (UNDP) to provide socioeconomic context.

Under-five mortality was chosen as the dependent variable because it is the definitive and most comprehensive indicator of child health and survival. All indicators were visually represented through thematic maps (i.e., visual mapping) generated using Visme software (version 6.8.1), applying color scales to illustrate the status of each country. The generation of these thematic maps was conducted purely for descriptive purposes – specifically, to visually illustrate and geographically classify the distribution of child health indicators across continents, rather than to achieve a separate inferential scientific goal. Indices were compared to highlight disparities, with all methods following ecological study standards to ensure consistency, validity, and reliability of findings.

### Research ethics statement and informed consent

Ethical approval was not required for this study because it is based exclusively on anonymized, publicly available secondary data from the Multiple Indicator Cluster Survey (MICS6), conducted and disseminated by UNICEF and national partners, which follow international ethical and data protection standards.Informed consent was not required for this study.

## Statistical analysis

Parametric assumptions, including homoscedasticity and the normality of residuals (assessed via the Shapiro-Wilk test), were rigorously tested prior to inferential analysis. To address potential collinearity, variance inflation factors (VIF) and tolerance values were evaluated before executing the regression models; no independent variable exceeded commonly accepted thresholds (VIF>10 or tolerance<0.10). A multiple linear regression was subsequently performed to evaluate how specific health indicators and social determinants (HDI, vaccination coverage, low birth weight, safe drinking water, and skilled birth care) predict under-five mortality, which was established as the primary dependent variable. Data were expressed as mean ± standard deviation (SD) or n (%). The statistical significance level was set at a 2-sided p value of ≤0.05. All statistical analyses were conducet using IBM-SPSS software, version 26.0.

The 95 % confidence intervals (CIs) for the standardized regression coefficients (*β*) were calculated by multiplying the lower and upper bounds of the CIs for the unstandardized coefficients by the standardization factor, which is the ratio between the standard deviations of the predictor and the dependent variable. Specifically, the standardized beta β is given by *β=*B*(SDx/SDy), where B is the unstandardized coefficient, SDx is the standard deviation of the predictor, and SDy is the standard deviation of the dependent variable. The lower and upper limits of the standardized beta CI were obtained by applying the same transformation to the corresponding limits of the unstandardized coefficient CI, i.e., CI*β*=CI_B_*(SDx/SDy), ensuring an accurate interpretation of the statistical uncertainty in standardized scale.

This study employed pairwise deletion (available-case analysis) to handle missing data. Pairwise deletion uses all cases with complete information for each pair of variables, increasing data use compared to listwise deletion and helping to preserve statistical power in correlation and regression.

## Results

Of the 72 countries and territories initially assessed for eligibility, 13 were excluded: eight due to their data collection status was marked “not completed”, four because they relied on previously published data, and one because the UNICEF report was unavailable. Consequently, 58 countries and territories with sufficient data completeness were included in the inferential analyses (Table 1). An additional 19 was excluded from the visual-analysis subset – 16 for missing indicator data and three for absent Human Development Index (HDI) information. Therefore, 39 countries and territories met the criteria for all predetermined variables and were retained for the visual mapping (Supplementary Figure 1; Supplementary Table 1).

### Under-five mortality risk

The regression model demonstrated that access to safe drinking water (*β* = −0.63; 95 % CI: −1.11 to −0.14; p=0.013, VIF=3.227; Tolerance=0.310) and HDI (*β* = −101.18; 95 % CI: −175.03 to −27.32; p=0.008, VIF=3.575; Tolerance=0.280) were significantly and inversely associated with under-five mortality risk, indicating that improvements in these determinants correspond to substantial reductions in under-five mortality rates. Other covariates – low birth weight, adequate skilled childbirth care, and vaccination coverage – did not achieve statistical significance in this model (p>0.05 for all) (Figure 1, **Panel A**).

**Figure 1:**
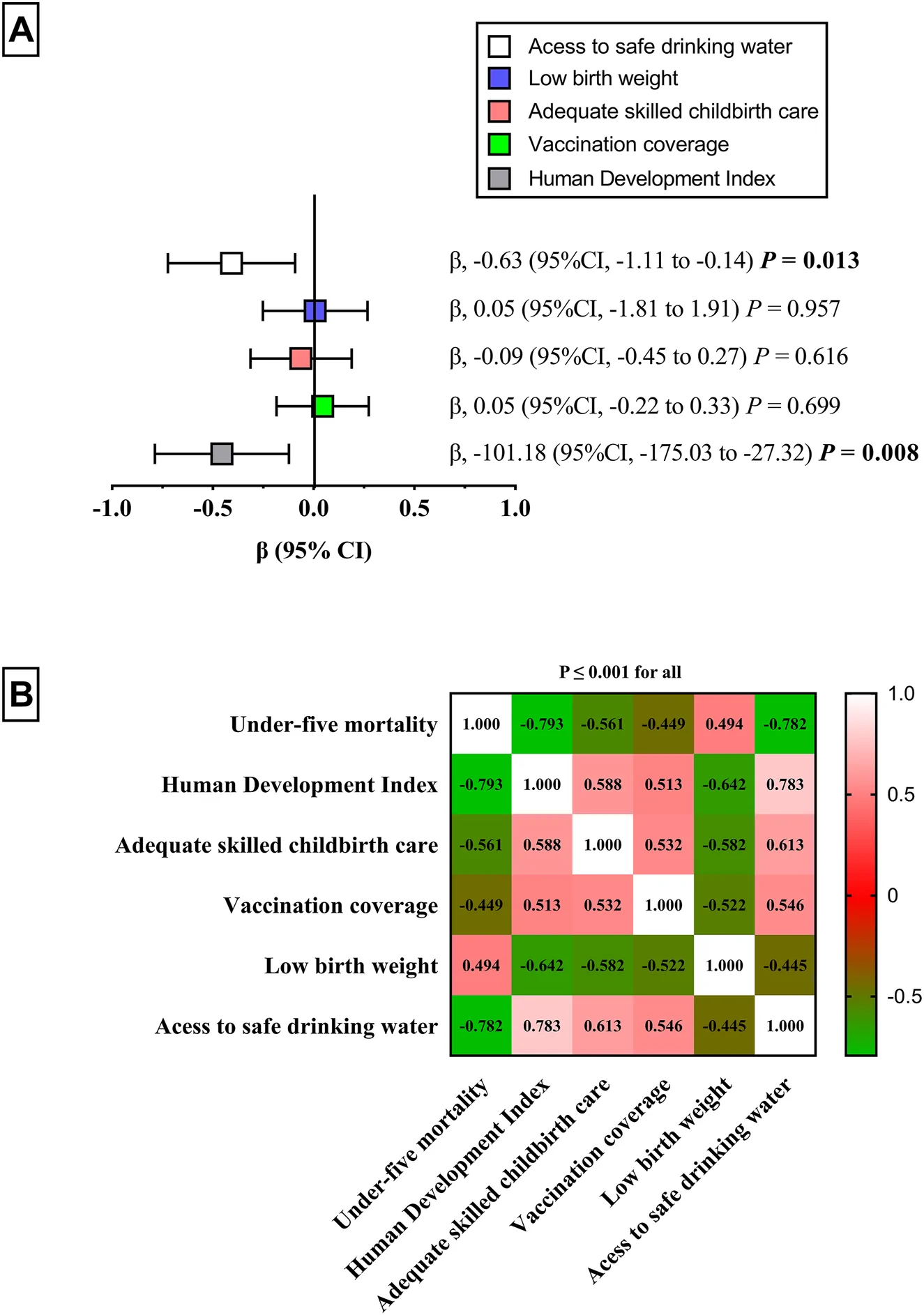
Multiple linear regression of under-five mortality risk and Pearson correlation among health indicators and social determinants, according to MICS6 and HDI. Panel A. Forest plot demonstrates the standardized regression coefficients (*β*) with 95 % confidence interval (CI) for under-five mortality risk. Standardized regression coefficients (*β*) were obtained by multiplying the unstandardized coefficients by the ratio between the standard deviations of the predictor (HDI) and the outcome. The 95 % confidence intervals for the standardized coefficients were derived by applying the same transformation to the lower and upper bounds of the confidence intervals of the unstandardized coefficients. Panel B. Heatmap demonstrating the correlation’ strength with respective values. All variables showed p≤0.001.

### Pearson correlation

Pearson correlation coefficients revealed strong negative associations between under-five mortality and both HDI (r = −0.793) and access to safe drinking water (r = −0.782), while moderate negative correlations were observed with adequate skilled childbirth care (r = −0.561) and vaccination coverage (r = −0.449). The correlation matrix also highlights interrelations among predictors, most notably the positive correlation between HDI and access to safe drinking water (r = 0.783), and HDI with vaccination coverage (r = 0.513). All correlations among variables were statistically significant (p<0.001 for all entries) (Figure 1, **Panel B**).

### Visual mapping

About the visual mapping, 39 countries administering the complete MICS6 Under-Five questionnaire (plus HDI) were included in the analysis. The geographic distribution comprised Africa (n=17), Asia (n=13), Europe (n=1), Oceania (n=5), and the Americas (n=3). Within the Americas, countries were further classified as Central America (n=2) and South America (n=1).

### Under-five mortality

Regarding under-five mortality rates per 1,000 live births, Africa encompassed the five countries with the highest rates, with the Central African Republic ranking foremost at 99 deaths per 1,000 live births (Figure 2). In contrast, Serbia, representing the sole European nation in the sample, demonstrated the lowest observed rate at nine deaths per 1,000 live births.

**Figure 2:**
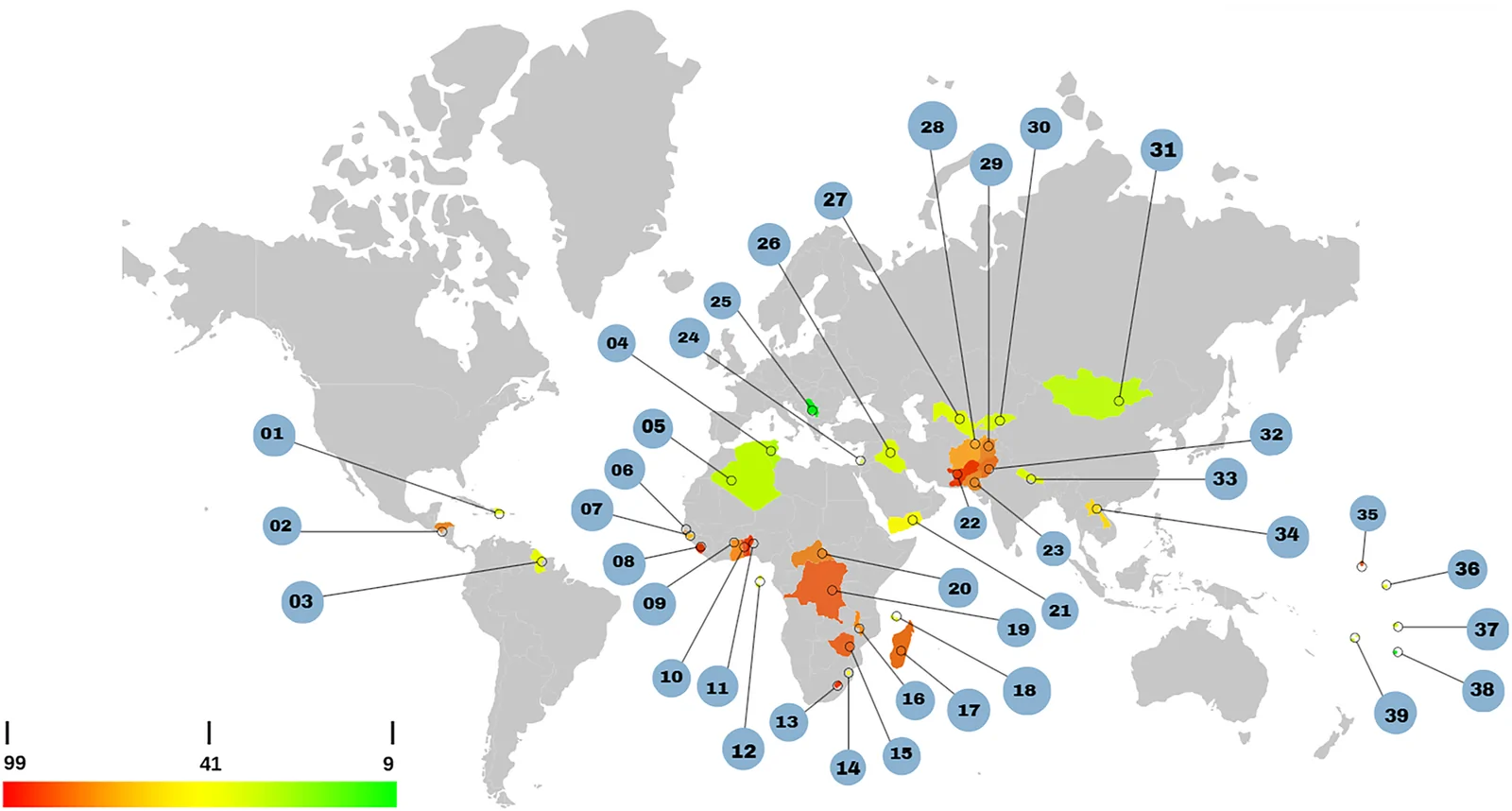
Under-five mortality. Descriptive analysis measured in MICS6 as the number of deaths of children under five years per 1,000 live births, reported by mothers based on birth and survival history. 1. Dominican Republic, 2. Honduras, 3. Guyana, 4. Tunisia, 5. Algeria, 6. Gambia, 7. Guinea-Bissau, 8. Sierra Leone, 9. Ghana, 10. Togo, 11. Benin, 12. São Tomé and Príncipe, 13. Lesotho, 14. Eswatini, 15. Zimbabwe, 16. Malawi, 17. Madagascar, 18. Comoros, 19. Democratic Republic of the Congo, 20. Central African Republic, 21. Yemen, 22. Pakistan (Balochistan), 23. Pakistan (Sindh), 24. State of Palestine, 25. Serbia, 26. Iraq, 27. Kyrgyzstan, 28. Afghanistan, 29. Pakistan (Khyber Pakhtunkhwa), 30. Uzbekistan, 31. Mongolia, 32. Pakistan (Punjab), 33. Nepal, 34. Lao People’s Democratic Republic, 35. Kiribati, 36. Tuvalu, 37. Samoa, 38. Tonga, 39. Fiji.

In Asia, considerable heterogeneity in under-five mortality was observed, particularly within Pakistan, which is regionally composed of four administrative provinces and showed pronounced disparities. Punjab Province exhibited a markedly elevated rate (69 per 1,000 live births), while Khyber Pakhtunkhwa Province showed a substantially lower rate (39 per 1,000 live births), corresponding to a difference of 43 % between these provinces (Figure 2).

### Human development index

The HDI – incorporated for its global relevance, though not a standard MICS6 indicator – revealed marked contrasts: Serbia (Europe) had the highest HDI (0.802), while Yemen (Asia, 0.435) and Central African Republic (Africa, 0.404) were lowest in their respective continents (Figure 3). For Pakistan, all provinces were aggregated at an HDI of 0.544. In South America, Guyana presented an HDI of 0.713, with the highest in Central America observed in the Dominican Republic (0.767) (Figure 3).

**Figure 3:**
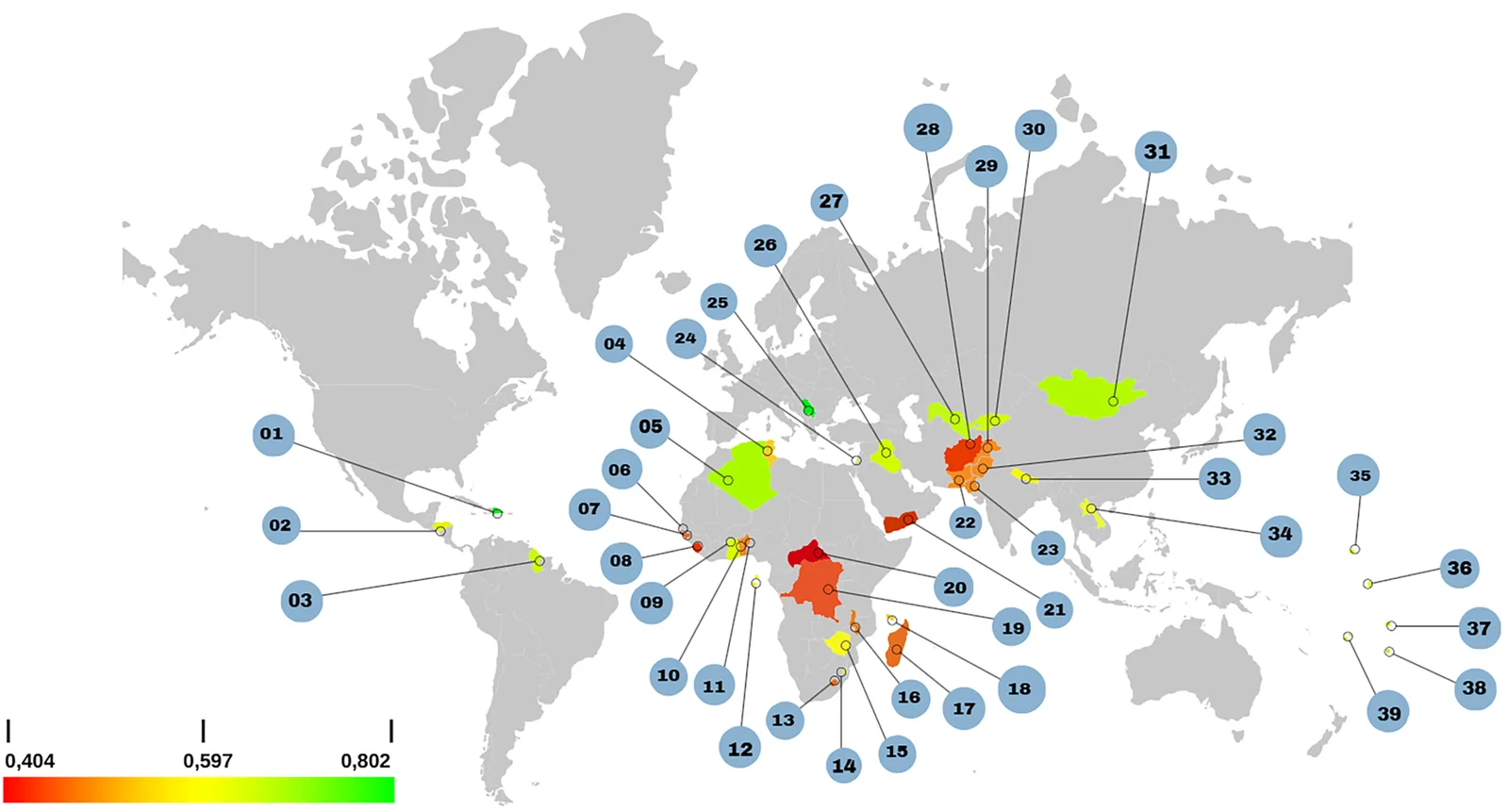
Human development index. Composite index measuring average achievement in three basic dimensions – health (life expectancy at birth), education (mean years of schooling and expected years of schooling), and standard of living (gross national income per capita). 1. Dominican Republic, 2. Honduras, 3. Guyana, 4. Tunisia, 5. Algeria, 6. Gambia, 7. Guinea-Bissau, 8. Sierra Leone, 9. Ghana, 10. Togo, 11. Benin, 12. São Tomé and Príncipe, 13. Lesotho, 14. Eswatini, 15. Zimbabwe, 16. Malawi, 17. Madagascar, 18. Comoros, 19. Democratic Republic of the Congo, 20. Central African Republic, 21. Yemen, 22. Pakistan (Balochistan), 23. Pakistan (Sindh), 24. State of Palestine, 25. Serbia, 26. Iraq, 27. Kyrgyzstan, 28. Afghanistan, 29. Pakistan (Khyber Pakhtunkhwa), 30. Uzbekistan, 31. Mongolia, 32. Pakistan (Punjab), 33. Nepal, 34. Lao People’s Democratic Republic, 35. Kiribati, 36. Tuvalu, 37. Samoa, 38. Tonga, 39. Fiji.

### Access to safe drinking water services

When considering access to safe drinking water services, the lowest rates in Africa were found in the Democratic Republic of the Congo (33.2 %), Central African Republic (37.5 %), Madagascar (41.0 %), and Sierra Leone (59.5 %) (Figure 4). In Asia, Afghanistan (68.8 %), Yemen (72.8 %), and the Lao People’s Democratic Republic (78.2 %) had the lowest access proportions. Highest access rates included Algeria (94.1 %) and Tunisia (85.9 %) in Africa, Pakistan Punjab (95.9 %) and Palestine (99.2 %) in Asia, and Tuvalu (Oceania) with the overall maximum (99.4 %) (Figure 4).

**Figure 4:**
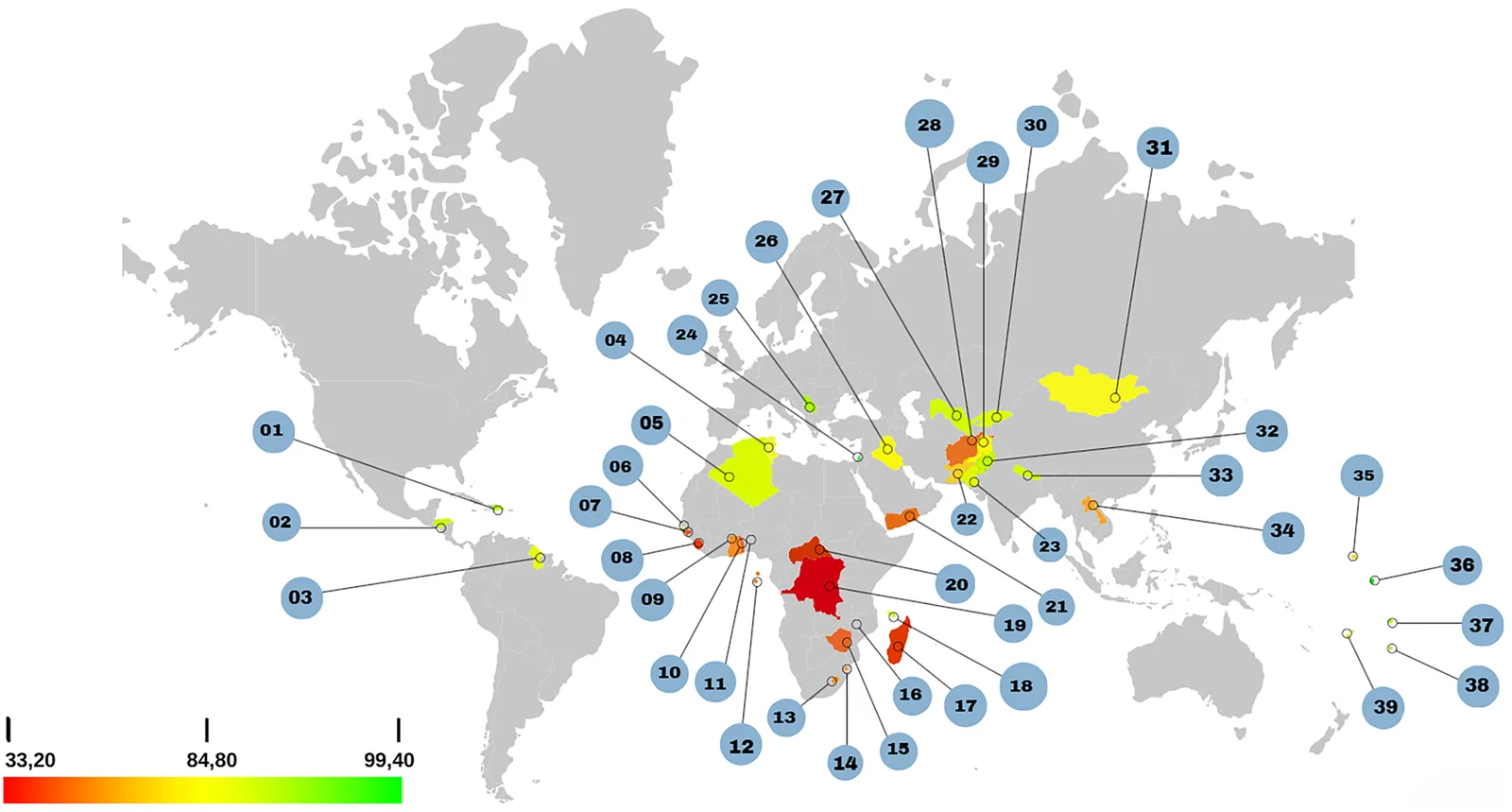
Access to safe drinking water services. Assessed according to WHO/UNICEF joint monitoring program criteria, by inquiring about household sources of drinking water and treatment practices, classifying water sources as safely managed or improved. 1. Dominican Republic, 2. Honduras, 3. Guyana, 4. Tunisia, 5. Algeria, 6. Gambia, 7. Guinea-Bissau, 8. Sierra Leone, 9. Ghana, 10. Togo, 11. Benin, 12. São Tomé and Príncipe, 13. Lesotho, 14. Eswatini, 15. Zimbabwe, 16. Malawi, 17. Madagascar, 18. Comoros, 19. Democratic Republic of the Congo, 20. Central African Republic, 21. Yemen, 22. Pakistan (Balochistan), 23. Pakistan (Sindh), 24. State of Palestine, 25. Serbia, 26. Iraq, 27. Kyrgyzstan, 28. Afghanistan, 29. Pakistan (Khyber Pakhtunkhwa), 30. Uzbekistan, 31. Mongolia, 32. Pakistan (Punjab), 33. Nepal, 34. Lao People’s Democratic Republic, 35. Kiribati, 36. Tuvalu, 37. Samoa, 38. Tonga, 39. Fiji.

### Complete vaccination coverage

For complete vaccination coverage among children under five years of age (Figure 5), over half of the participating countries attained rates above 50 %, with the overall median at 68.3 % – as seen in Tuvalu, Oceania. The lowest rates were recorded in Africa, notably in the Central African Republic (13.2 %) and Guinea-Bissau (19.3 %), whereas Zimbabwe had the highest African coverage at 84.5 %. Guyana, representing South America, reported 70.6 %, while coverage in Central America ranged from 62.1 % in the Dominican Republic to 77.6 % in Honduras. In Asia, Uzbekistan had the highest coverage (94.0 %) and Baluchistan, Pakistan, the lowest (26.9 %). The greatest overall observed coverage was in Fiji (Oceania) at 99.8 % (Figure 5).

**Figure 5:**
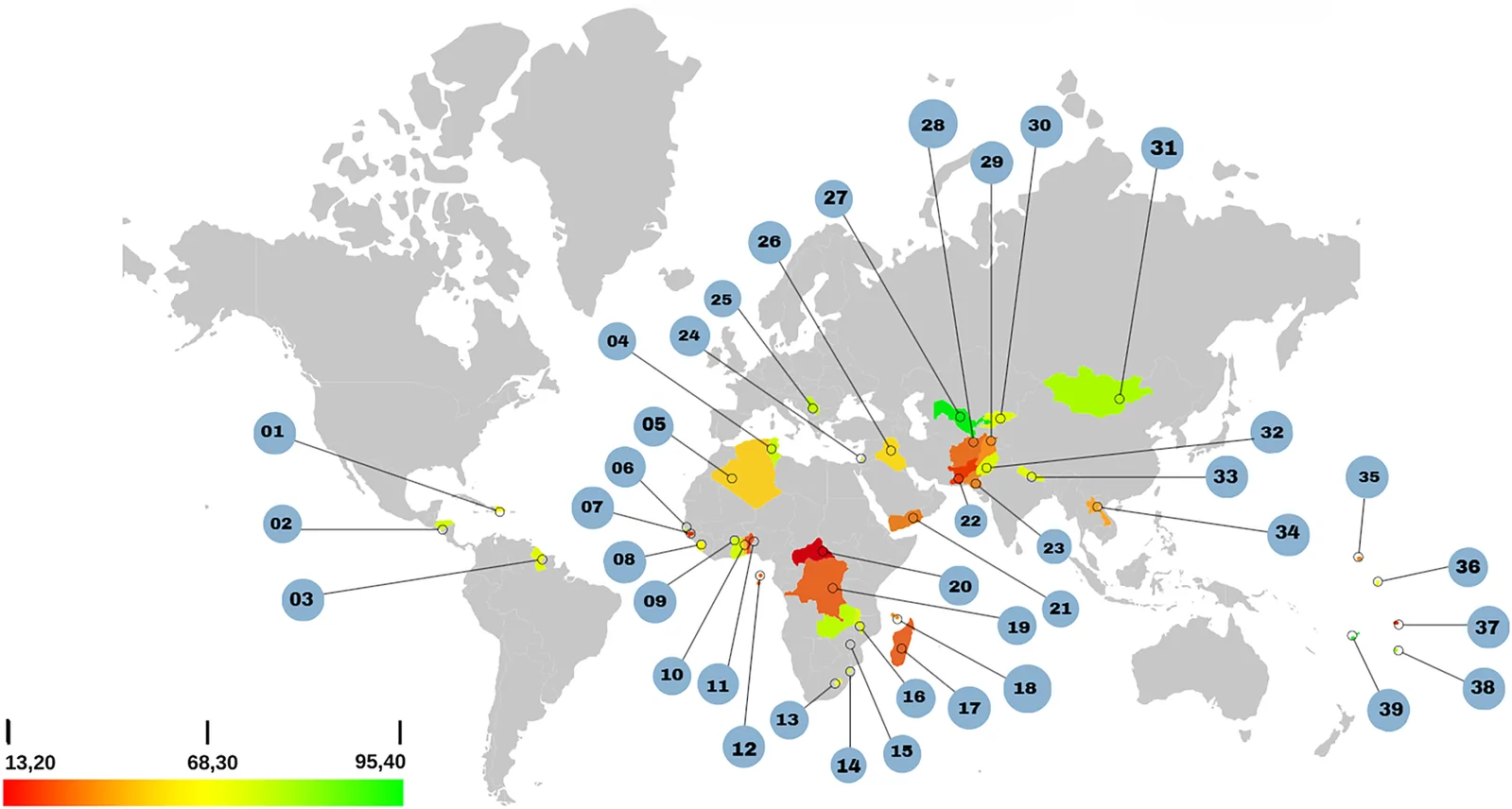
Complete vaccination coverage. Descriptive analysis determined by assessing whether children under five have received all doses of recommended vaccines per national immunization schedules, using vaccination cards and/or caregiver reports. 1. Dominican Republic, 2. Honduras, 3. Guyana, 4. Tunisia, 5. Algeria, 6. Gambia, 7. Guinea-Bissau, 8. Sierra Leone, 9. Ghana, 10. Togo, 11. Benin, 12. São Tomé and Príncipe, 13. Lesotho, 14. Eswatini, 15. Zimbabwe, 16. Malawi, 17. Madagascar, 18. Comoros, 19. Democratic Republic of the Congo, 20. Central African Republic, 21. Yemen, 22. Pakistan (Balochistan), 23. Pakistan (Sindh), 24. State of Palestine, 25. Serbia, 26. Iraq, 27. Kyrgyzstan, 28. Afghanistan, 29. Pakistan (Khyber Pakhtunkhwa), 30. Uzbekistan, 31. Mongolia, 32. Pakistan (Punjab), 33. Nepal, 34. Lao People’s Democratic Republic, 35. Kiribati, 36. Tuvalu, 37. Samoa, 38. Tonga, 39. Fiji.

### Prevalence of low birth weight

Examining the prevalence of severe low birth weight (Figure 6), the most elevated rates were found in Asia – Sindh (Pakistan) at 18.1 %, Yemen at 17.7 %, and Baluchistan (Pakistan) at 11.6 %. In Africa, the Democratic Republic of the Congo registered 7.1 %, and Algeria the lowest continental rate (0.8 %). Among Central American countries, Honduras reported 1.2 % and the Dominican Republic 0.6 %. The lowest study rates were seen in Kyrgyzstan and Uzbekistan (both Asia, 0.4 %), Tonga (Oceania, 0.3 %), and Serbia (Europe, 0.2 %) (Figure 6).

**Figure 6:**
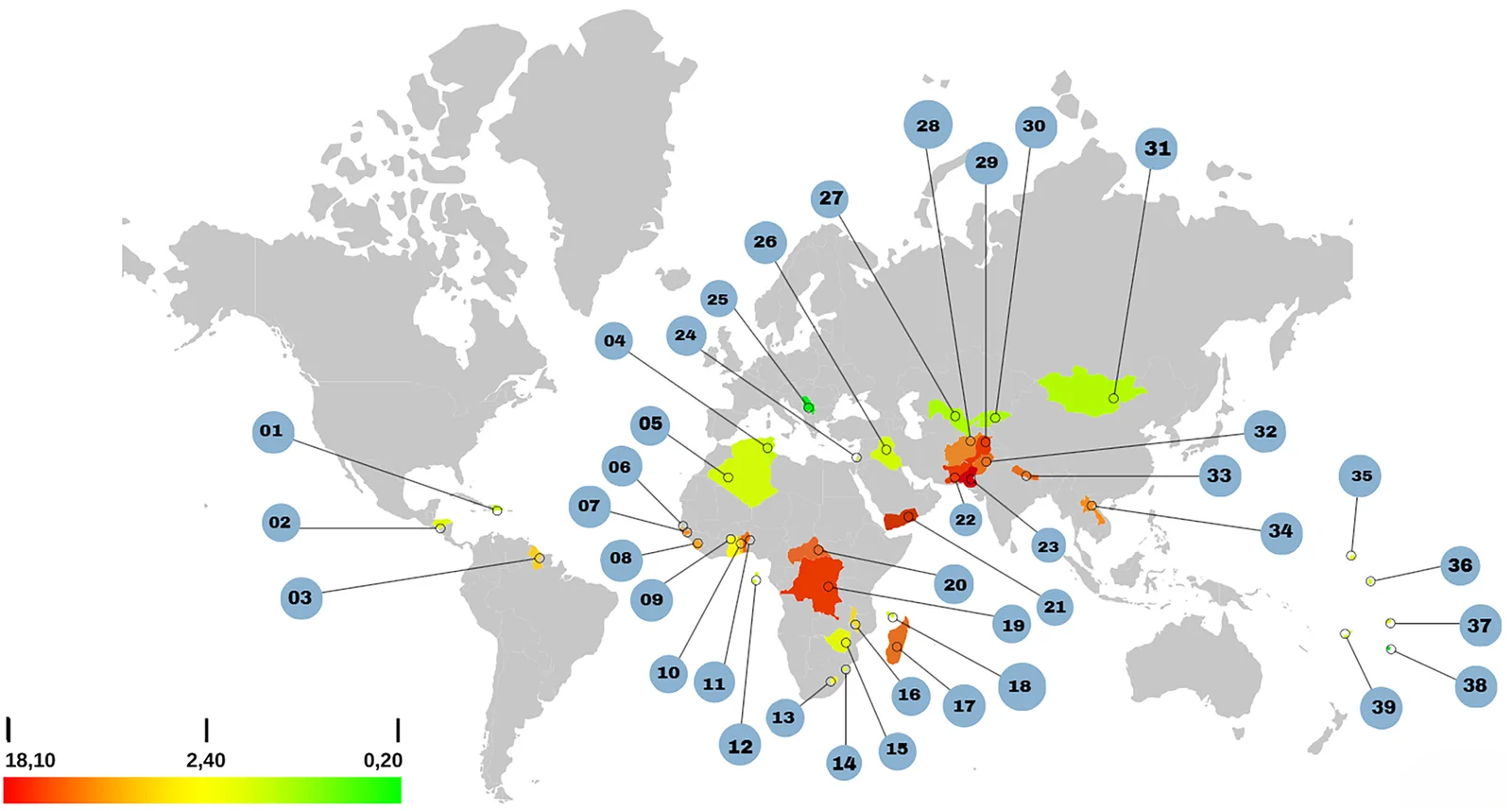
Prevalence of low birth weight. Calculated from birth weight data recorded on health cards or reported by mothers, with low birth weight defined as less than 2,500 g. 1. Dominican Republic, 2. Honduras, 3. Guyana, 4. Tunisia, 5. Algeria, 6. Gambia, 7. Guinea-Bissau, 8. Sierra Leone, 9. Ghana, 10. Togo, 11. Benin, 12. São Tomé and Príncipe, 13. Lesotho, 14. Eswatini, 15. Zimbabwe, 16. Malawi, 17. Madagascar, 18. Comoros, 19. Democratic Republic of the Congo, 20. Central African Republic, 21. Yemen, 22. Pakistan (Balochistan), 23. Pakistan (Sindh), 24. State of Palestine, 25. Serbia, 26. Iraq, 27. Kyrgyzstan, 28. Afghanistan, 29. Pakistan (Khyber Pakhtunkhwa), 30. Uzbekistan, 31. Mongolia, 32. Pakistan (Punjab), 33. Nepal, 34. Lao People’s Democratic Republic, 35. Kiribati, 36. Tuvalu, 37. Samoa, 38. Tonga, 39. Fiji.

### Adequate skilled childbirth care

Concerning skilled birth attendance (Figure 7), most investigated countries demonstrated high rates, with the continental median represented by Lesotho (86.6 %, Asia). However, Pakistan (Baluchistan, 49.9 %), Madagascar (46.0 %), and the Central African Republic (42.9 %) all evidenced rates below 50 %. The highest rates were observed in Fiji (Oceania), Kyrgyzstan (Asia) – both 99.8 %, and Serbia (Europe, 99.9 %) (Figure 7).

**Figure 7:**
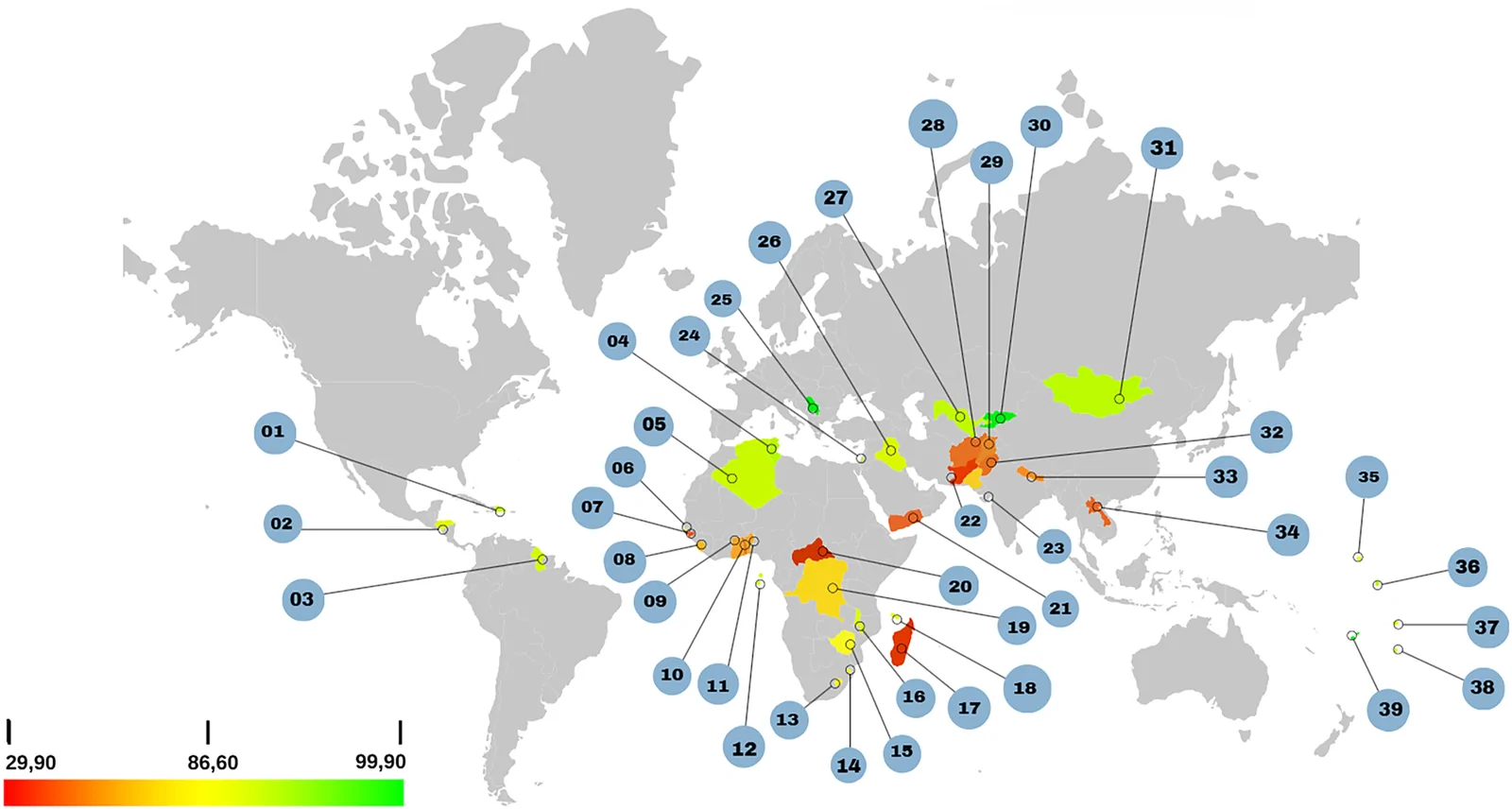
Adequate skilled childbirth care. Defined as the proportion of births attended by skilled health personnel (e.g., doctors, nurses, midwives), sourced from maternal recall in survey modules concerning recent births. 1. Dominican Republic, 2. Honduras, 3. Guyana, 4. Tunisia, 5. Algeria, 6. Gambia, 7. Guinea-Bissau, 8. Sierra Leone, 9. Ghana, 10. Togo, 11. Benin, 12. São Tomé and Príncipe, 13. Lesotho, 14. Eswatini, 15. Zimbabwe, 16. Malawi, 17. Madagascar, 18. Comoros, 19. Democratic Republic of the Congo, 20. Central African Republic, 21. Yemen, 22. Pakistan (Balochistan), 23. Pakistan (Sindh), 24. State of Palestine, 25. Serbia, 26. Iraq, 27. Kyrgyzstan, 28. Afghanistan, 29. Pakistan (Khyber Pakhtunkhwa), 30. Uzbekistan, 31. Mongolia, 32. Pakistan (Punjab), 33. Nepal, 34. Lao People’s Democratic Republic, 35. Kiribati, 36. Tuvalu, 37. Samoa, 38. Tonga, 39. Fiji.

## Discussion

The present study revealed substantial heterogeneity across 39 developing countries, highlighting divergent child health outcomes shaped by distinct socioeconomic and geopolitical contexts. Access to safe drinking water and HDI emerged as critical determinants inversely associated with under-five mortality, as shown by multivariable regression and correlation analyses, underscoring their foundational roles in early childhood health disparities.

Social determinants of health are structural factors that shape living and health conditions – including governance, policies, cultural norms, and socioeconomic hierarchies – and they profoundly influence health trajectories, particularly in early childhood [7], 28], 29]. Marked inequalities in socioeconomic, political, religious, and cultural domains expose certain groups to elevated risks and vulnerabilities, substantially impacting early childhood development [30], [31], [32]. These determinants operate as root causes of health disparities by influencing access to resources, opportunities, and environments essential for healthy growth.

Population segmentation into distinct socioeconomic strata and social hierarchies creates uneven progress among countries, exacerbating disparities in access to essential resources for healthy development [33]. This is reflected in recent widespread declines in the HDI, with over 90 % of countries experiencing drops in 2020 or 2021, as reported by the UNDP. Nations with lower HDI scores often face heightened human insecurity, marked by reduced interpersonal trust, increased societal polarization, and diminished individual agency, particularly among lower-income groups who experience limited control and institutional distrust [34]. These intersecting factors amplify vulnerabilities in early childhood development and highlight the complex structural influences behind health disparities.

The 2024 Human Development Report provides updated empirical evidence reinforcing the structural and social determinants framework [34]. It documents continued global declines in human development indices, accentuated political polarization, and increased economic concentration among high-income countries. Serbia – uniquely representing Europe in this analysis – registered the highest HDI (0.802), distinctly surpassing African, Asian, American, and Oceanian nations. These contrasting outcomes illustrate the urgent need for nuanced public policies tailored to each country’s historical, geographic, and cultural context, with particular attention to African countries facing persistent development challenges and the lowest HDI scores.

Regional-specific findings align with global health literature: African countries exhibited the poorest outcomes across multiple indicators, reflecting entrenched systemic disadvantages manifesting as high under-five mortality rates, low vaccination coverage, limited access to potable water, and elevated prevalence of low birth weight. Africa remains profoundly affected by the enduring legacy of exploitative colonialism, which consolidated socioeconomic imbalances through extractive economic systems, centralized governance structures, and persistent social hierarchies [35]. These historical processes have perpetuated structural inequalities that continue to hinder equitable development and contribute to persistent disparities in health and socioeconomic conditions across the continent [36].

The sociopolitical context and early childhood health are deeply linked, with individual capacity and development profoundly influenced by the surrounding environment [37], 38]. Social justice provides a vital foundation for individuals to build meaningful lives by ensuring basic living conditions [39]. The gap between current living conditions and human capacity development necessitates public policies centered on equity and social justice, viewed through an intersectional lens. Such policies should promote social participation and strive to provide all children with equitable opportunities for healthy, comprehensive development, aiming to overcome systemic inequities [40].

In Asia, the highest under-five mortality rates were observed in politically conflicted settings, including Afghanistan and Pakistan’s Punjab province, with rates of 50.4 and approximately 69 per 1,000 live births respectively, reflecting severe socioeconomic destabilization. Conversely, lower under-five mortality rates from 2017 to 2023 were reported in Palestine (14.2 per 1,000) and Mongolia (19 per 1,000). Although Palestine ranked second-best in the continent according to MICS6 data from 2019–2020, the ongoing Gaza conflict has severely exacerbated child mortality through direct violence and the secondary effects of war, including compromised sanitation, water scarcity, malnutrition, and increased incidence of preventable diseases such as diarrhea [2].

Vaccination coverage has been critical to improving child development, directly contributing to mortality reduction by preventing deaths from vaccine-preventable diseases [41]. Although progress has been made in African immunization programs, most countries have not met WHO target of 90 % coverage in at least 30 % of member states [42]. This study confirms that coverage below 50 % remains common in many African and Asian countries, particularly where internal conflicts disrupt essential services, including immunization. The Central African Republic exemplifies such a crisis, with vaccination coverage for children under one year falling to approximately 51 %, well below global targets [43]. Given limited literature and ongoing challenges, urgent coordinated efforts are essential to ensure immunization access in conflict-affected and crisis settings.

In Oceania, vaccination dropout in Samoa reached 26.8 %, driven in part by anti-vaccination movements that spread misinformation, exacerbating the 2019 measles outbreak which infected over 5,700 people and caused 83 deaths, predominantly among children under five years of age [44]. This crisis highlighted the severe public health consequences of reduced vaccine coverage and the critical need for effective vaccination campaigns and countermeasures against misinformation.

Countries experiencing sociopolitical instability, including all Pakistani provinces, Yemen, the Democratic Republic of Congo, and the Central African Republic, demonstrate the highest prevalence of severe low birth weight among children under five, directly attributable to entrenched food insecurity arising from socioeconomic inequalities, economic crises, and disrupted supply chains. These conditions manifest as inadequate quantity and quality of nutrition essential for optimal fetal and postnatal growth [45], diverging substantially from Sustainable Development Goal 2 targets to eradicate hunger and ensure year-round access to safe, nutritious food. Low birth weight and resultant stunting precipitate long-term functional deficits, motor skill impairments, and cognitive delays, underscoring the need for integrated nutritional interventions embedded within broader social determinants frameworks to mitigate intergenerational health inequities [24].

Qualified delivery assistance – encompassing comprehensive antenatal, intrapartum, postpartum, and neonatal care – remains critically deficient in many study nations, particularly across Sub-Saharan Africa and conflict zones, where approximately 70 % of global maternal deaths occurred in 2020 [46], with crisis-affected countries exhibiting rates exceeding twice the worldwide average (551 vs. 223 per 100,000 live births) [47]. This disparity heightens risks of maternal and neonatal complications, infections, and mortality, necessitating scalable models of skilled birth attendance to align with WHO benchmarks for universal coverage [48]. Concurrently, suboptimal access to potable water, sanitation, and hygiene – recognized as a fundamental human right – correlates strongly with elevated diarrheal disease burdens, malnutrition, and child mortality, particularly in low-resource African and Asian settings [49], demanding multisectoral biomedical-social interventions to uphold equity and avert preventable morbidity [7], 24].

The marked regional disparities in early childhood health call for multisectoral strategies that integrate evidence-based interventions such as health workforce development, infrastructure enhancement, vaccination campaigns, and improvements in education and healthcare services. Addressing broader social determinants through socioeconomic initiatives is essential. Specifically, expanding the use of MICS6 to a country as diverse and large as Brazil would provide critical insights into regional inequities and guide tailored policies to improve child health outcomes nationwide.

We cannot disregard that the exclusion of 33 countries with incomplete or unavailable information for all indicators selected for the visual mapping may have introduced a selection bias, as the 39 included countries may not fully represent all developing countries participating in MICS6, particularly those with weaker information systems. However, this approach was necessary to ensure comparability across indicators, and the descriptive characteristics of the included countries (e.g., geographic distribution and HDI levels) suggest that they still encompass a broad range of contexts.

In addition, although the study was designed as an intercontinental analysis, the final sample of 39 countries is predominantly composed of African and Asian settings, with only one European country (Serbia) and one South American country (Guyana), which may limit the generalizability and interpretability of the comparative conclusions to other regions, reinforcing that the findings should be interpreted with caution and not assumed to be globally representative.

This study has several limitations. First, using secondary data from MICS6 and relying on reports may limit representativeness. Second, aggregated population data prevented control of individual risk factors, local inequalities, or specific public policies, restricting external validity and inference in vulnerable subgroups. Third, although pairwise deletion uses all cases with complete information, helping to preserve statistical power in correlation and regression analyses, the assumption that missingness is completely at random may not fully hold, given the cross-national nature of the MICS6 data, and this approach may introduce bias if missingness is related to structural or unobserved factors. Therefore, further advanced methods for handling missing data (such as multiple imputation) would be desirable in future studies. The study’s main strength is its integrated analysis of multidimensional child health indicators from MICS6 combined with HDI evaluation, allowing comprehensive insight into regional disparities across diverse sociopolitical and economic contexts. This quantitative, comparative approach, complemented by an in-depth discussion on structural social determinants, provides evidence to guide targeted, context-sensitive public policies promoting equity in child development and addressing population-specific vulnerabilities.

## Conclusions

The findings indicate that access to safe drinking water and higher Human Development Index (HDI) scores are significantly and inversely related to under-five mortality risk, particularly in Africa. These results underscore the importance of contextual social and health determinants in shaping child mortality patterns across countries. The quantitative approach enabled a nuanced understanding of both direct and interrelated effects, highlighting the need to allocate resources and implement integrated, evidence-based public health strategies to improve childhood survival worldwide. Further prospective studies are needed to deepen understanding of these factors across diverse settings and to inform clinical and multidisciplinary practices that advance equity, social justice, and continuous monitoring within a comprehensive framework.

## Supplementary Material

Supplementary Material
