# Supplementary material for: Multiple indicator cluster survey (MICS) related to early childhood health: an ecological intercontinental analysis
Source: Open Med (Wars). 2026 Oct 2;21(1):20261495. doi: 10.1515/med-2026-1495 (PMC13629718; doi:10.1515/med-2026-1495)
Supplement: Supplementary file 1 [file j_med-2026-1495_suppl_001.docx]

**Supplemental Online Content**

**Supplementary Figure 1. Flowchart of countries and territories in the study.** The data were divided into inferential and (N=58) and descriptive analyses (N=39).

| **Supplementary Table 1. Reported indicators of under-five mortality, complete vaccination coverage, adequate skilled childbirth care, prevalence of low birth weight, and basic access to safe drinking water services in developing countries participating in MICS6** **included in mapping (N=39).** | | | | | | |
| --- | --- | --- | --- | --- | --- | --- |
|  | **Human Development Index** | **Under-five mortality** | **Adequate skilled childbirth care** | **Complete vaccination coverage** | **Prevalence of low birth weight** | **Access to safe drinking water services** |
| **Africa (N=17), mean ± SD** | 0.544 **±** 0.078 | 56.5 **±** 25.1 | 80.9 **±** 18.0 | 55.8 **±** 23.6 | 3.2 **±** 2.1 | 69.4 **±** 18.3 |
| Malawi | 0.512 | 56.0 | 96.4% | 72.6% | 2.6% | 74.2% |
| São Tomé and Príncipe | 0.618 | 14.0 | 96.8% | 41.5% | 1.4% | 77.2% |
| Comoros | 0.558 | 36.0 | 97.2% | 57.0% | 1.9% | 91.9% |
| Eswatini | 0.597 | 41.0 | 93.4% | 77.0% | 1.3% | 78.2% |
| Benin | 0.525 | 80.0 | 80.8% | 27.6% | 5.9% | 72.2% |
| Tunisia | 0.576 | 17.0 | 99.5% | 79.1% | 0.6% | 85.9% |
| Central African Republic | 0.404 | 99.0 | 42.9% | 13.2% | 7.0% | 37.5% |
| Guinea-Bissau | 0.483 | 51.0 | 53.8% | 19.3% | 3.8% | 59.8% |
| Madagascar | 0.501 | 59.0 | 46.0% | 33.0% | 6.0% | 41.0% |
| Lesotho | 0.514 | 76.2 | 86.6% | 68.8% | 2.5% | 79.4% |
| Gambia | 0.500 | 57.0 | 82.7% | 83.2% | 2.7% | 84.8% |
| Zimbabwe | 0.593 | 65.0 | 86.0% | 84.5% | 1.6% | 63.6% |
| Algeria | 0.745 | 19.0 | 98.8% | 61.0% | 0.8% | 94.1%  68.1% |
| Togo | 0.539 | 71.0 | 69.4% | 48.5% | 2.8% |  |
| Ghana | 0.632 | 56.0 | 78.9% | 78.1% | 2.4% | 79.4% |
| Sierra Leone | 0.477 | 94.0 | 81.6% | 69.4% | 3.7% | 59.5% |
| Democratic Republic of the Congo | 0.479 | 70.0 | 85.2% | 35.0% | 7.1% | 33.2% |
| **Asia (N=12), mean ± SD** | 0.606 ± 0.101 | 36.3 ± 17.0 | 82.1 ± 17.0 | 61.6 ± 22.9 | 6.1 ± 6.0 | 87.1 ± 9.6 |
| Pakistan (Sindh) | 0.544 | 46.0 | 82.7% | 43.8% | 18.1% | 90.4% |
| Pakistan (Punjab) | 0.544 | 69.0 | 76.4% | 76.5% | 6.4% | 95.9% |
| Pakistan (Balochistan) | 0.544 | 53.0 | 49.9% | 26.9% | 11.6% | 79.6% |
| Pakistan (Khyber Pakhtunkhwa) | 0.544 | 39.0 | 76.5% | 45.8% | 7.1% | 86.8% |
| State of Palestine | 0.715 | 14.2 | 99.7% | 86.0% | 0.6% | 99.2% |
| Nepal | 0.602 | 28.0 | 77.2% | 70.2% | 6.5% | 95.4% |
| Iraq | 0.686 | 26.0 | 95.6% | 58.1% | 1.0% | 85.7% |
| Mongolia | 0.739 | 19.0 | 99.3% | 86.9% | 0.5% | 82.5% |
| Kyrgyzstan | 0.692 | 20.0 | 99.8% | 76.7% | 0.4% | 92.6% |
| Uzbekistan | 0.727 | 25.0 | 99.2% | 94.0% | 0.4% | 95.0% |
| Yemen | 0.455 | 41.0 | 60.9% | 38.3% | 14.7% | 72.8% |
| Afghanistan | 0.478 | 55.0 | 67.5% | 36.6% | 5.6% | 68.8% |
| **Europe (N=1)** | 0.803 ± 0.022 | 12.5 ± 4.9 | 99.6 ± 0.5 | 83.9 ± 3.7 | 0.6 ± 0.7 | 97.5 ± 2.8 |
| Serbia | 0.802 | 9.0 | 99.9% | 81.0% | 0.2% | 99.1% |
| **Central America (N=2), mean ± SD** | 0.694 ± 0.103 | 26.5 ± 7.8 | 96.6 ± 3.6 | 69.8 ± 11.0 | 0.9 ± 0.4 | 97.0 ± 1.9 |
| Honduras | 0.621 | 21.0 | 94.1% | 77.6% | 1.2% | 95.7% |
| Dominican Republic | 0.767 | 32.0 | 99.2% | 62.1% | 0.6% | 98.4% |
| **Oceania (N=5), mean ± SD** | 0.689 ± 0.054 | 28.0 ± 19.7 | 95.7 ± 5.0 | 65.2 ± 30.5 | 1.1 ± 0.6 | 95.1 ± 7.9 |
| Fiji | 0.730 | 18.0 | 99.8% | 95.4% | 1.2% | 97.6% |
| Tonga | 0.745 | 11.0 | 98.3% | 93.5% | 0.3% | 99.0% |
| Samoa | 0.707 | 20.0 | 88.9% | 26.8% | 1.4% | 98.6% |
| Tuvalu | 0.641 | 30.0 | 99.5% | 68.3% | 0.7% | 99.4% |
| Kiribati | 0.624 | 61.0 | 91.9% | 42.1% | 2.0% | 81.0% |
| **South America (N=2), mean ± SD** | 0.722 ± 0.012 | 22.0 ± 4.2 | 98.0 ± 0.6 | 49.2 ± 30.2 | 1.7 ± 1.3 | 96.0 ± 2.0 |
| Guyana | 0.713 | 25.0 | 97.6% | 70.6% | 2.7% | 94.6% |
| Suriname | 0.730 | 19.0 | 98.4% | 27.9% | 0.8% | 97.5% |
